# A Review on Bioflocculant-Synthesized Copper Nanoparticles: Characterization and Application in Wastewater Treatment

**DOI:** 10.3390/bioengineering11101007

**Published:** 2024-10-10

**Authors:** Nkanyiso C. Nkosi, Albertus K. Basson, Zuzingcebo G. Ntombela, Nkosinathi G. Dlamini, Rajasekhar V. S. R. Pullabhotla

**Affiliations:** 1 Biochemistry and Microbiology Department, Faculty of Science, Agriculture, and Engineering, University of Zululand, P/Bag X1001, KwaDlangezwa 3886, South Africa; 2 Chemistry Department, Faculty of Science, Agriculture, and Engineering, University of Zululand, P/Bag X1001, KwaDlangezwa 3886, South Africa

**Keywords:** bioflocculant, Cu nanoparticles, biosynthesis, characterization, application

## Abstract

Copper nanoparticles (CuNPs) are tiny materials with special features such as high electric conductivity, catalytic activity, antimicrobial activity, and optical activity. Published reports demonstrate their utilization in various fields, including biomedical, agricultural, environmental, wastewater treatment, and sensor fields. CuNPs can be produced utilizing traditional procedures; nevertheless, such procedures have restrictions like excessive consumption of energy, low production yields, and the utilization of detrimental substances. Thus, the adoption of environmentally approachable “green” approaches for copper nanoparticle synthesis is gaining popularity. These approaches involve employing plants, bacteria, and fungi. Nonetheless, there is a scarcity of data regarding the application of microbial bioflocculants in the synthesis of copper NPs. Therefore, this review emphasizes copper NP production using microbial flocculants, which offer economic benefits and are sustainable and harmless. The review also provides a characterization of the synthesized copper nanoparticles, employing numerous analytical tools to determine their compositional, morphological, and topographical features. It focuses on scientific advances from January 2015 to December 2023 and emphasizes the use of synthesized copper NPs in wastewater treatment.

## 1. Introduction

The accessibility of excellent-quality potable water is crucial for human survival, but the release of toxic waste from industries is a significant source of groundwater pollution [1]. Traditional wastewater treatment techniques, including adsorbents, reverse osmosis, ion exchange, and electrostatic precipitation, have drawbacks such as being costly, less efficient, and with poor recyclability. Although sustainable technologies have been developed, their usage is limited due to their material properties, including cost and potential health hazards [2,3].

Recent studies underline the significance of various sorbents for water purification. For instance, Yudaev et al. [4] stated that modified chitosan can be effectively utilized as a sorbent due to its enhanced hydrophobic properties which make it suitable for oil spill cleanup applications. Chitosan’s biodegradable nature further supports its role as an eco-friendly alternative for removing contaminants from water sources. Additionally, textiles have emerged as promising sorbents for wastewater treatment. Zaarour and Liu [5] provide an extensive review of textile-based sorbents that highlights their potential when combined with activated carbon or other functional materials to enhance pollutant removal efficiencies significantly while maintaining low operational costs. The incorporation of textiles into water treatment processes not only improves adsorption capabilities but also promotes sustainability by utilizing existing materials effectively. These developments highlight the potential for integrating innovative sorbent materials into existing water treatment frameworks to enhance efficacy while addressing environmental concerns associated with traditional methods. However, the research lacks extensive field testing, which raises questions about its effectiveness in diverse real-world conditions and over extended periods. Nonetheless, it does not sufficiently address the scalability of these technologies or potential challenges in their implementation at an industrial level.

With that being said, bioflocculants are regarded as a viable alternative for conventional coagulants utilized in treating wastewater as well as industrial uses. Bioflocculants are naturally occurring biopolymers produced by specific microorganisms like bacteria, fungi, and algae [6]. Typically, bioflocculants consist of polysaccharides, proteins, or a combination of both [7,8]. These natural polymers have a substantial molecular weight and contain different functional groups in their structure [9]. As a result, they can effectively interact with particles in liquids and aid in the process of flocculation. Bioflocculants are created from materials that are non-toxic, biodegradable, and eco-friendly, since they originate from natural resources and do not constitute any harmful chemicals [10,11]. However, the production of bioflocculants can be limited by low yields and high costs, which hinder their scalability for industrial applications. Additionally, their flocculating efficiency may vary based on wastewater characteristics, and thorough assessments of their long-term environmental impacts are still needed [12,13]. These limitations have led researchers to explore alternative technologies for wastewater treatment.

The use of nanoparticles may potentially solve these problems and address the presence of pollutants in wastewater, such as pesticides, organic–inorganic pollutants, and heavy metals. Nanoparticles are substances that have sizes that range from 1 to 100 nm, possessing distinctive traits encompassing magnetic, optical, and electrical properties [14]. Nanoparticle matters are widely utilized in several fields, including environmental detection, biomedical science, clothing, and cosmetics. Nanoparticles exhibit strong adsorption, catalytic activities, and reactivity resulting from their smaller dimensions and high atom proportion at the surface. They also have a stratified structure and rotatable band edges for optimized catalysis, a direct bandgap, low cost, a high optical absorption coefficient, and low toxicity [15].

Copper NPs are applied in wastewater treatment as a result of distinctive features, which include their diminutive size, low production cost, significant surface area, and effectiveness in removing pollutants [16]. Copper nanoparticles possess antibacterial properties that impede the multiplication of microbes, fungi, viruses, and algae. However, concerns regarding their environmental toxicity and long-term impacts on ecosystems remain inadequately addressed in many studies [17].

Over the years, numerous approaches have been employed to fabricate nanoparticles, depending on their nature and type [18]. In general, both are two kinds of strategies known as “top-down and bottom-up”. The technique used from the top down involves decreasing large substances to obtain nanoparticles, whereas the bottom-up procedure encompasses the production of NPs from element chemicals [19]. These methodologies have been employed in different fabricating processes, entailing physical, chemical, and biological approaches [20]. Physical procedures include lithography, pyrolysis, vapor deposition, crushing, grinding, attrition, and ball milling. Chemical methods include chemical vapor deposition, hydrothermal and solvothermal methods, the sol-gel method, thermal decomposition, microwave-assisted synthesis, ultrasonic-assisted synthesis, reduction by photocatalysis, electrochemical approaches, and gas-phase processes [21].

These methods are distinguished by their ability to produce nanomaterials with a uniform size distribution and homogeneity [22]. They are, however, characterized by substantial energy consumption in fabrication, high costs, and low productivity. They include time-consuming synthesis techniques, and a certain number of the solvents utilized for nanoparticle formation are harmful and hazardous, leading to the generation of perilous NPs and the development of natural contamination [20]. The hazardous nature of chemically produced NPs, along with their intrinsic volatility, renders them economically and environmentally unfeasible, severely limiting their biological uses [23]. This has additionally resulted in the development of cost-effective and environmentally friendly biological techniques, such as “green” nanotechnology [24].

Greener NP production is a possible area of research in the field of sustainable nanotechnology [25]. This procedure is less toxic, eco-friendly, efficient, and cost-efficient in comparison to conventional physical and chemical procedures. Biologically sourced agents, such as plants, bacteria, algae, and fungi, are commonly employed in the green synthesis of NPs [26]. Green-synthesized NPs are particularly successful at eliminating and retrieving contaminants from effluents, without causing the degradation of organic pollutants [27]. This makes them ideal for purifying wastewater for reuse and recycling, and they have the potential to address numerous global water quality issues.

Metallic copper nanoparticles are gaining popularity as a result of their distinctive qualities, like a large surface-to-volume proportion, tiny dimension, elevated melting points, sustainability, and affordability [28]. CuNPs have strong antibacterial activity against a broad range of bacteria, including *Salmonella enterica*, *Escherichia coli*, *Listeria monocytogenes*, and *Staphylococcus aureus*, when compared to silver and iron nanoparticles [29]. CuNPs are more effective than iron oxide nanoparticles in fighting against multidrug resistance biofilm-producing pathogenic bacteria [30]. Furthermore, CuNPs have high mobility in solution, and researchers have discovered that they are good at removing heavy metals, organic pollutants, inorganic, and microorganisms [31].

Many studies have synthesized nanoparticles using plants, which include *Tilia* [32], *Punica granatum* [33], *Broccoli* [34], *Celastrus paniculatus* [35], *Cissus vitiginea* [36], *Curcuma longa* [37], *Azadirachta indica* [38], *Cinnamon bark* [39], *Ginger* [40], *Cynodon dactylon* [41], *Hyptis suaveolens* [42], *Schefflera arboricola* [43], and *Nigella sativa* [44]. However, few studies have detailed the synthesis of nanoparticles using microbial bioflocculants. For example, Dlamini et al. [45], Adebayo-Tayo et al. [46], Ntombela et al. [47], and Tsilo et al. [48] synthesized iron, silver, zinc oxide, and copper NPs using bioflocculants, respectively. Therefore, there is a dearth of information concerning the biosynthesis of Cu nanoparticles using microbial bioflocculants. Thus, this review is intended to explore the most recent advances from the year January 2015 to December 2023 in the production of Cu nanoparticles employing microbial bioflocculants. The characterization of the biosynthesized CuNPs and their application are also reviewed in this article.

## 2. Different Methods for CuNP Synthesis

### 2.1. Chemical Approaches

Chemical procedures are one of the most commonly employed for the production of CuNPs. This is because chemical methods allow for high precision in the size, shape, and crystal phase of the NPs [49]. However, the cost of these approaches is high and results in low-yield production [49]. Chemical procedures such as hydrothermal synthesis, and electrochemical, chemical, and sonochemical reduction are found in the creation of CuNPs [50]. In chemical reduction, reducing factors such as hydrazine, ascorbic acid, or sodium borohydride are typically employed [51].

The study by Nguyen et al. [52] employed precursors such as NaH_2_PO_2_, N_2_H_4,_ and NaBH_4_ and polymeric protecting agents to synthesize Cu nanoparticles. The authors acquired copper nanoparticles ranging in size from 3 to 9 nm. They also perceived that the inadequacy of liquid in NP production showed a tiny advantageous influence on the firmness of the acquired NPs.

Alonso et al. [53] utilized 135 mg, 1.0 mmol of dehydrated Cu (II) Cl, and 14 mg, 2.0 mmol of lithium power in air argon to create Cu nanoparticles. By modifying the copper salt dosage, reducing factor, the solution’s pH, and temperature synthesis, the researchers produced nanoparticles of varying sizes, shapes, and activities.

The traditional chemical methods of synthesizing NPs require high pressure, energy, temperature, and poisonous chemicals, which are disadvantageous to nature and human well-being. Therefore, the replacement of chemical methods with green synthesis technology is becoming increasingly necessary. Green procedure is a potential and eco-approachable procedure for generating materials with individual properties.

### 2.2. Physical Methods

Physical approaches for synthesizing nanoparticles involve the use of physical processes to produce nanoparticles. Evaporation–condensation, laser ablation, high-energy ball milling, and thermal decomposition are considered the most significant physical synthesis methods [54]. These methods offer several advantages over chemical synthesis, including the lack of solvent contamination and the achievement of uniform nanoparticle distribution [55]. However, poor control over the morphology of the nanomaterials, the presence of unexpected toxic ions in the final products, and difficulty in regulating the size and shape of the NPs are some of the drawbacks of nanoparticle formation [49].

Crisan et al. [56] utilized evaporation–condensation to produce CuNPs. The authors revealed that the extremely small nanoparticles, ranging from 6.2 to 21.5 nm and 1.23 to 1.88 nm were achieved.

Kaabipour and Hemmati [57] conducted a study on the synthesis of CuNPs using laser ablation. The authors reported the sizes between 20 and 50 nm to be suitable for nanoparticle formation.

Physical synthesis approaches for nanoparticle formation have some inherent disadvantages. For example, these technologies often demand a significant energy input and specialized equipment, making them prohibitively expensive and restricting their scalability for large-scale production. Furthermore, many physical synthesis procedures entail the use of harsh chemicals or intense reaction conditions, which may result in the development of toxic byproducts and pose safety concerns to researchers and the environment. These limitations emphasize the need for alternate, more sustainable synthesis processes, such as green synthesis.

The green production of NPs has generated considerable attention regarding its environmentally friendly methodology, compatibility with living systems, cost-effectiveness, adaptability, and potential for medical applications. By utilizing natural resources and employing mild reaction conditions, this synthesis technique minimizes the ecological impact typically associated with conventional nanoparticle production, which often involves hazardous substances and produces harmful waste. Furthermore, the biocompatible characteristics of nanoparticles synthesized through green methods make them suitable for various medical purposes, such as targeted drug administration, imaging techniques, and combating microbial infections. The versatility of green synthesis allows for the precise manipulation of nanoparticle properties, facilitating customization for specific applications in fields like catalyst development, electronics, advanced diagnostics, and wastewater purification. Thus, the emphasis of this review revolves around the production of Cu nanoparticles utilizing microbial bioflocculants.

### 2.3. Biological Methods for CuNP Synthesis

There are various green synthesis methods for producing nanoparticles that use plants, microbes, and other organisms. All of these methods are considered to be both safe and effective [58,59]. These methods are given in detail below.

#### 2.3.1. Green Synthesis of Copper Nanoparticles Using Plants

**Figure 1:**
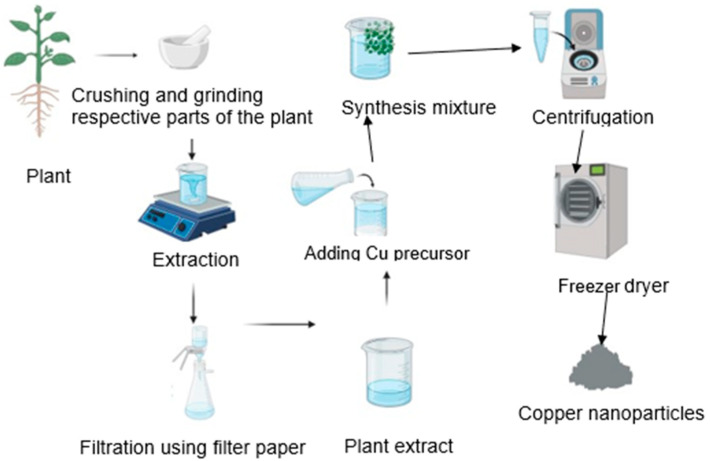
Graphic illustration depicting the mediated production of CuNPs utilizing plant extract [77]. Created with BioRender.com. https://www.biorender.com (accessed on 27 April 2024).

Plants provide various benefits for Cu nanoparticle production, particularly their ready availability, safe handling, and the presence of a diverse compass of metabolites which are useful in the elimination processes [60]. Interestingly, certain plants are capable of synthesizing nanoparticles as they absorb metal ions that exceed tolerable concentrations. Dissimilar plant parts like stems, leaves, roots, flowers, and other components can be utilized in the synthesis procedure. When employing plant-based materials, the metal salt is compounded with plant extracts, and the reaction is allowed to complete at room temperature for a period of 1 to 3 h (Figure 1).

Alao et al. [61] used *Kigelia Africana* fruit extract to synthesize copper nanoparticles. About 0.25 M aqueous solution of copper acetate was prepared and stored at a dark room temperature. Subsequently, 50 mL of the prepared copper acetate solution was combined with 25 mL of the *Kigelia Africana* fruit extract in a 250 mL Erlenmeyer flask. The solution was continuously agitated for 3 h with the assistance of a magnetic stirrer, ensuring thorough mixing. After stirring, the solution was left undisturbed for 24 h without any sort of light. To separate the CuNPs, the mixture was centrifuged at 10,000 rpm for 15 min. The resultant precipitate was then rinsed with purified water to eliminate any remaining biological extract. The rinsed precipitate was transferred to an oven for 4 h at 80 °C to obtain the as-synthesized CuNPs.

Rajeshkumar and collaborators published a report in which they utilized the leaf extract of *Cissus arnotiana* to produce CuNPs. The researchers observed that the produced copper NPs displayed an irregular and spherical shape, with a diameter size ranging between 60 and 90 nm [62].

Vidovix et al. [63] fabricated CuNPs employing *Punica granatum* leaf extract. Copper NP formation was observed to have a spherical shape and an average size of 20.33 nm.

Kaur et al. [64] detailed the production of copper nanoparticles utilizing the peel extract of *Punica granatum* and observed the particle sizes ranging from 15 to 20 nm. The produced CuNPs exhibited strong antibacterial effects against opportunistic pathogens such as *Micrococcus luteus* MTTC 1809, *Pseudomonas aeruginosa* MTTC 424, *Salmonella enterica* MTCC 1253, and *Enterobacter aerogenes* MTCC 2823.

Zangeneh et al. [65] conducted a study on CuNP synthesis using *Falcaria vulgaris* leaf extract, which demonstrated significant cytotoxicity, antioxidant properties, antifungal and antibacterial effects, as well as wound-healing capabilities. The findings suggested that the synthesized CuNPs could be beneficial for therapeutic and industrial purposes.

Ahmed et al. [66] published the successful formation of CuNPs from the leaf extracts of *Camelia sinensis*. Nieto-Maldonado et al. [67] also demonstrated the successful fabrication of CuNPs, employing Gardenia *jasminoides* plant extract.

Other noteworthy outcomes have been achieved in the production of NPs utilizing dissimilar parts of plants, such as *Enicostemma axillare* leaf extract for copper oxide nanoparticles [68], seedless date extract [69], and a fruit extract of *Myristica fragrans* [70].

Numerous other plants have also been employed in the production of CuNPs, including *P*. *granatum* seed extract [71], *Allium noeanum* leaf extract [72], *C*. *spinose* fruit extract [73], *Azadirachta indica* leaf extract [74], *Piper retrofractum Vahl* extract [75], *Cissus vitiginea* [76], and seedless dates [69], among various others.

#### 2.3.2. Green Synthesis of Copper NPs Using Bacteria

**Figure 2:**
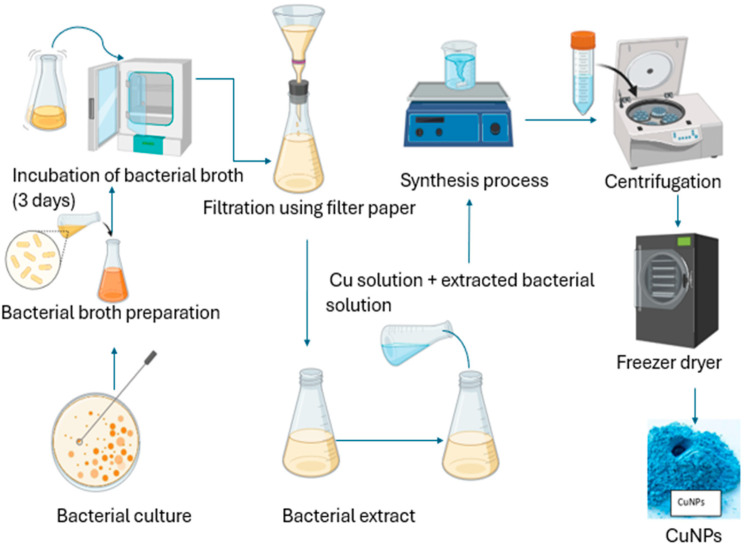
Graph depicting the fabrication of copper NPs utilizing bacteria [77]. Created with BioRender.com. https://www.biorender.com (accessed on 27 April 2024).

The desire for an easy, cheap, and dependable procedure for NP formation led to the utilization of bacteria to produce copper nanoparticles [78]. Synthesis techniques, utilizing bacteria, have arisen as an environmentally friendly, hygienic, and feasible substitute for traditional approaches [79]. Over the past few years, bacteria have been exploited to synthesize respective types of nanoparticles, including copper nanoparticles [80]. Through either an intercellular or extracellular process (Figure 2), bacteria have successfully generated diverse materials with captivation shapes and nanoscale sizes [77,81]. The utilization of bacteria for nanoparticle production holds immense potential due to several advantages [82]. These include a quick reproduction cycle, convenient culturing, favorable experimental conditions, remarkable stability to synthesize nanoparticles externally, and straightforward genetic modification [83]. Microorganisms have been observed to adapt and survive in environments with hazardous metals by converting toxic metal ions to less harmful forms like metal sulfide or oxides [84]. Extensive research supports the notion that bacteria can withstand high levels of dangerous metals by revolutionizing detrimental metal ions into safe metal oxides [85]. Additionally, bacteria have been found to produce various thiol-containing compounds as a response to oxidative stress. These substances protect metal oxide nanoparticles from oxidation during the bacterially induced production of NPs [86].

Noman et al. [87] conducted the research using *Escherichia* sp. for the production of Cu nanoparticles. The strain of microorganisms was introduced into the Luria–Bertani broth and cultured in a rotating stirring mixer at 200 rpm and 22 °C. A volume of 1 mM was achieved after 24 h by adding Cu sulphate and 5H_2_O. The prepared combination was then incubated at 22 °C for 24–48 h on a rotating agitator at 150 rpm. To serve as a control, heat-killed bacterial strain or Luria–Bertani medium without bacterial strain was maintained, with the addition of 1 mM CuSO_4._5H_2_O. The combined solution’s color altered from sky-blue to bluish-green, indicating the production of CuNPs. In the UV area, a point at 325.89 was detected, further indicating the harmonization of copper nanoparticles.

Bhagat et al. [88] synthesized copper nanoparticles using microbes such as *Phormidium cyanobacterium*, *Escherichia coli*, *Morganella morganii*, *Serratia* sp., *Stereum hirsutum*, and *Aspergillus niger* strain STA9. These microorganisms were reported to fabricate Cu nanoparticles of different sizes that range between 2 and 398.2 nm.

Bukhari et al. [89] reported the formation of CuNPs using *Streptomyces* sp. To synthesize, 15 mL of 1 mM of CuSO_4_ was poured into 15 mL of *Streptomyces* sp., after which the mixture was placed in an agitate incubator at 30–32 °C, for 24 h, 150 rpm. The color modification from greenish blue to drab green was observed, confirming the successfully synthesized CuNPs. The formed nanoparticles were further verified by utilizing a UV–Vis spectrograph, after which maxima at 550 nm were observed.

Ghasemi and co-workers reported the formed copper nanoparticles using *Morganela morganii*. To synthesize, 50 mL of the bacterium solution was poured into the growth of the production medium containing 50 µL of CuSO_4_.H_2_O. The broth was shaken for 24 h, at 37 °C and 150 rpm. Later on, a color alteration from blue-green to greenish was observed. CuNPs were obtained after centrifuging the solution. The produced NPs were further confirmed by conducting procedures such as XRD, FESEM, XAFS, UV–Vis, and EDS. The examination demonstrated that the copper nanoparticles synthesized through biological means possessed a sphere shape and a diameter of 10 nm. Additionally, the antibacterial effects of the NPs were examined, and the findings indicated their potential as effective agents against a range of harmful bacteria. In summary, the employment of various bacteria in the production of copper NPs has made significant contributions to this field of research [85].

#### 2.3.3. Green Synthesis of Copper Nanoparticles Using Fungi

**Figure 3:**
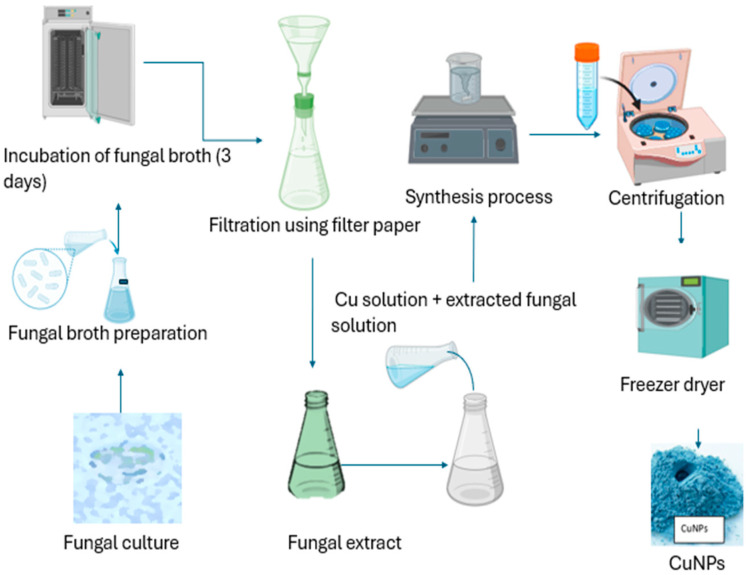
Graphical depiction of biosynthesis of copper NPs utilizing different fungi [77]. Created with BioRender.com. https://www.biorender.com (accessed on 27 April 2024).

Several species of fungi have been employed to fabricate CuO_2_ and other metal NPs in recent decades [90]. Fungi, in comparison to other bacteria, have a high capacity for producing NPs [91]. In contrast to bacteria, fungi can withstand shaking, pressure flow, and other circumstances in a bioreactor or other development chamber. Microorganism cell-free extracts operate as reducing, catalytic, or capping agents in the biogenic synthesis of NPs [92]. *Trichoderma* species produce an extensive wide range of bioactive metabolites, such as pyrenes, polyketides, terpenes, diketopiperazine, glycolipids, and several reductive enzymes that help in the synthesis of not only CuNPs but also Ag and ZnO NPs [93]. Fungi produce nanoparticles through both internal and external pathways. Nanoparticles formed within species of fungi may be smaller in size in comparison to those formed via the extracellular pathway, with greater dispersity and diameters [77]. Extracellular nanoparticle fabrication provides various advantages. The formation of nanoparticles may be devoid of cell components. Fungi release several enzymes that function as reducers and stabilizer agents in the formation of nanoparticles [93]. A variety of strains of fungi were used to produce metal oxide NPs, particularly copper nanoparticles [94]. The formation of CuNPs utilizing fungi is presented in Figure 3.

So, numerous different fungus species were researched in this endeavor, and it has been revealed that fungi are great choices due to the fact that they produce a high number of catalysts and thus were cheaper to deal with in the research conditions. Extracellular CuNPs have been discovered to be produced by *Penicillium aurantiogriseum*, *Penicillium citrinum*, and *Penicillium Tasmania* [95].

Saravanakumar et al. [96] testified the production of CuNPs utilizing *Trichoderma*. To produce these nanoparticles, a solution of Cu (NO_3_)_2_ 3H_2_O salt was introduced to a water extract free of mycelium and agitated at 40 °C before being put in a dim room overnight. The solution was then heated for 3 h at a thermal of 75–80 °C. As a result of this process, the solution changed color from blueish to dark blue, portraying the generation of Cu oxide NPs. The developed NPs went through various characterization techniques including spectrophotometry, X-ray diffraction (XRD), high-resolution scanning electron microscopy (HRSEM), particle size analysis, and transmission electron microscopy (TEM). According to the characterization results, the Cu nanoparticles displayed an average length of 110 nm and a spherical morphology. The FT-IR assessment further detected the existence of amide and another aromatic compound, indicating how such functional groups play a vital part in covering and supporting the copper NPs.

In the work of Cuevas et al. [90], it was tested that CuNPs were produced employing the white-rot fungus *Stereum hirsutum*. The researchers isolated a fungal extract without mycelium and showed it to a mixture consisting of numerous Cu salts, namely CuCl_2_, Cu (NO_3_)_2_, and CuSO_4_. This combination was placed in a shaker at a speed of 100 rpm for a duration of seven days, maintained in the dark at a temperature of 25 °C. During this process, the solution underwent a color change, turning into a chocolate shade, indicating the production of copper NPs. The structure, length, and physical composition were analyzed using various techniques such as UV–Vis spectroscopy, X-ray diffraction (XRD), transmission electron microscopy (TEM), and Fourier-transform infrared spectroscopy (FT-IR). The results revealed that the fabricated copper NPs exhibited a spherical shape with dimensions ranging from 5 to 20 nm.

El-Batal et al. [97] documented the production of CuO NPs using *Penicillium chrysogenum*, taking inspiration from biological processes. The researchers conducted various analyses, including UV–Vis spectroscopy, Fourier-transform infrared spectroscopy (FT-IR), X-ray diffraction (XRD), dynamic light scattering (DLS), transmission electron microscopy (TEM), scanning electron microscopy (SEM), and energy-dispersive X-ray spectroscopy (EDX), to verify the characteristics of the nanoparticles. According to the study, the copper nanoparticles synthesized through this biological method exhibit a globular shape with a mean width of 9.7 nm.

In one such study, the *Aspergillus niger* attained after the purification of biomass was used as a reductant agent for copper nanoparticle homogenization. A shift of color from light blue to dark blue was observed as a confirmation of the synthesized CuNPs [98]. A similar color change was observed when the white-rot fungus *Stereum hirsutum* and *Morganella* sp. were employed as reducing and protective agents, confirming the formation of nanoparticles [79].

#### 2.3.4. Green Synthesis of Copper Nanoparticles Using Algae

Algae, which are considered one of the earliest and most significant life forms on Earth, play a vital role in autotrophic processes by conducting over 50% of the planet’s photosynthesis [99]. Due to their abundance of biologically active substances, they are seen as an attractive option for serving as photosynthetic biorefineries, capable of producing a broad range of valuable products, including fuels [100]. Algae are recognized for their capability to consume high quantities of contaminants and convert them into more flexible forms. This remarkable feature has led to the consideration of algae as ideal organisms for producing bio-nanomaterials.

Algal members have become increasingly important in the formation of CuNPs. Gu et al. [101] reported the production of copper NPs utilizing *Cystoserira trinodis*. The diameter of the produced copper NPs ranged from 6 to 7.8 nm.

Arya et al. [102] specified that the formation of copper NPs using microalgae *Botrycoccus braunii* revealed a size length of 2 to 10 nm.

Bhattacharya et al. [103] employed an unusual approach by boiling the extract at 50 °C. A color shift from bright blue to coffee was detected as a symbol of CuNP formation. The synthesized CuNPs produced particle sizes of 3.6 nm.

**Figure 4:**
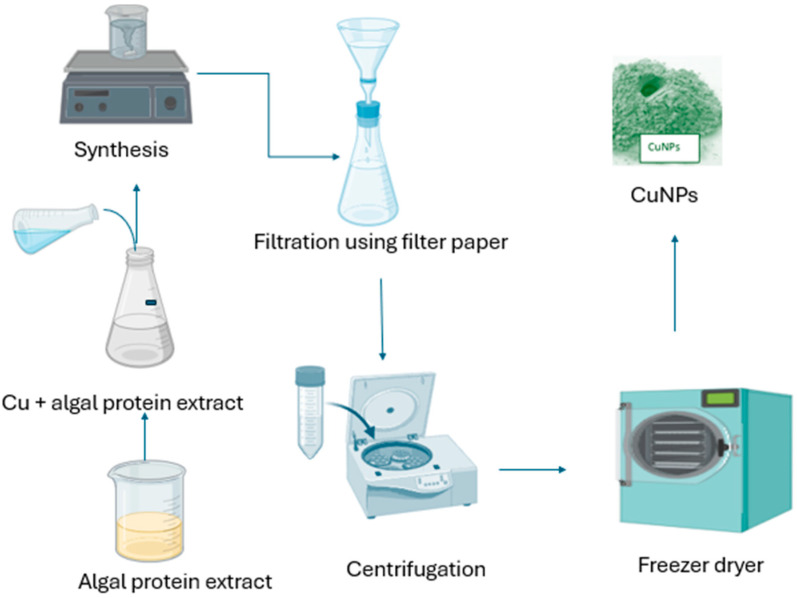
A visual depiction of the process of producing the algal extract proteins for the production of CuNPs utilizing various algae [77]. Created with BioRender.com. https://www.biorender.com (accessed on 27 April 2024).

Waris et al. [104] reported synthesized CuNPs using *Bifurcaria bifurcate* to exhibit microbicidal activity against *Enterobacter aerogenes* (Gram-negative) and *S*. *aureus* (Gram-positive) bacteria. The size of the produced copper NPs was detected to range from 5 to 45 nm. As a result, the multiple algal species liable for the capping and reducing method employing Cu as a promoter, along with the different organic apparatuses, are still not fully discovered (Figure 4) [77]. Therefore, it is crucial to pay attention to the production of CuNPs, utilizing green synthesis approaches to widen their biological usefulness.

#### 2.3.5. Green Synthesis of Copper Nanoparticles Using Microbial Bioflocculant

**Figure 5:**
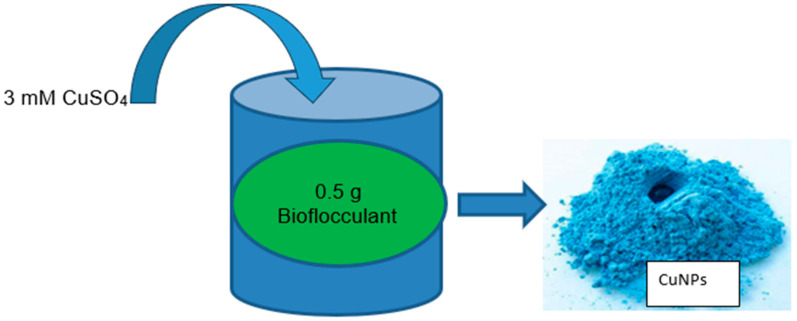
Illustration of biological production of copper NPs using bioflocculants [109].

The formation of NPs employing microbial bioflocculants is an intriguing field of study that merges nanotechnology and biotechnology [105]. Bioflocculants are natural polymers or compounds excreted by microorganisms, plants, or algae that possess the capability to induce the clumping or aggregation of particles within a liquid mixture [106]. A bioflocculant can act as a dipping and stabilizing agent for the development of NPs, including copper, silver, and iron nanoparticles [107,108]. This method is easy, inexpensive, and beneficial to the environment. Moreover, bioflocculants offer the ability to improve the properties and applications of NPs, such as flocculation, removal efficiency, antimicrobial activity, biocompatibility, stability, and optical, catalytic, and magnetic properties [45]. The precursors used are usually copper sulfate_,_ copper chloride, copper nitrate, and copper acetate which are mixed with a bioflocculant. The copper nanoparticles produced by such a method are illustrated in Figure 5. Nevertheless, few studies focus on the synthesis using bioflocculants. Moreover, most of these studies use plant-based bioflocculants for copper nanoparticle synthesis. Therefore, there is inadequate information on the generation of Cu NPs employing microbial bioflocculants, which, according to our knowledge, has only been studied by Bhattacharya et al. [103], Dlamini et al. [109], and Tsilo et al. [48].

Bhattacharya et al. [103] reported the fabrication of copper NPs employing microbial bioflocculants extracted from *Anabaena cylindrica*. In a 250 mL Erlenmeyer flask, 100 mL of 1 mM CuSO_4_ suspension was set on a magnetic hotplate stirrer. The extract algal (2.5 g/100 mL, 5 g/100 mL, and 10 g/100 mL) was supplemented dropwise onto the copper sulfate mixture while it was continuously agitated at a variable rotational rate at a thermal heat of 60 °C, respectively. The effects of pH on nanoparticle production were studied by altering the pH of the medium between 6.2 and 10.2. The solution’s shade shifted from sky-blue to indigo, signifying the development of copper NPs. The synthesized copper nanoparticles were repeatedly rinsed with deionized water before being centrifuged. The copper sulfate initial concentration solution ranged from 0.5 mM to 2.5 mM. The rinsed materials were air-dried in a heater at 100 °C for 24 h before being kept in a sealed bag for future usage.

Dlamini et al. [109] researched the biosynthesis of copper NPs employing bioflocculants isolated from *Alcaligenes faecalis* HCB2. The study involved adding 0.5 g of pure bioflocculant to a 200 mL liquid containing 3 mM CuSO_4_. The combination was stirred until it formed a uniform solution. The neck of the Erlenmeyer flask was covered with oil to prevent any interference from foreign materials. After that, the combination was kept undisturbed at ambient heat until 24 h. In this experiment, the bioflocculant solution was utilized as a control. The analysis of copper nanoparticles was observed after 24 h through physical and characterization. A blue solid was observed which confirmed the formation of CuNPs. The NPs were obtained employing a centrifuge that was set @ 4 °C and 8000 rpm for 30 min. The collected nanoparticles were then allowed to dry in a vacuum overnight and preserved for subsequent analysis.

Tsilo et al. [48] detailed the synthesis of copper nanoparticles utilizing the bioflocculant *Pichia kudriavzevii* attained from Kombucha Tea SCOBY. The production of the as-synthesized CuNPs was carried out by mixing a 200 mL solution of 3 mM of CuSO_4_ and 0.5 g of the produced bioflocculant in pure water. The solution was shaken at an ambient temperature for 10 min using an agitation incubator at a rotating rate of 200 rpm. The combination was left undisturbed in a dark area for twenty-four hours before the precipitate was assembled utilizing a centrifuge at 8000 rpm for 15 min at 4 °C. The researcher confirmed the synthesis of CuNPs by observing the color alteration of the solution from colorless to blue. The as-synthesized material was also confirmed by various characterization analytical methods. The control was copper sulfate solution with the absence of a bioflocculant. The formation of the synthesized CuNPs was placed in a safe place for future use. Some of the examples of the synthesis methods using chemical, physical, and biological approaches are indicated in Table 1.

## 3. Factors Affecting CuNP Synthesis

Although using microbial by-products to synthesize nanoparticles has the potential to be a good replacement for chemical and physical methods, the main drawback in applying the produced NPs is the inability to regulate both the size and shape of NPs. The primary goal of using bioflocculants in nanoparticle synthesis is to improve the production yield for industrial applications [47]. The size, design, and chemical structure of nanoparticles are affected by a variety of physiological conditions, such as heat, pH, metal-substrate dosage, nutrition, and biological catalysts. The optimization of these factors is required for the production of NPs with morphological features and chemical constituents. Some of the factors influencing nanoparticle biosynthesis are detailed below.

### 3.1. Effect of Precursor Concentration

It has been discovered that the precursor concentration utilized in the synthesis of CuNPs using microbial bioflocculants affects the characteristics and performance of the produced nanoparticles.

El-Saadony et al. [112] studied the effect of CuSO_45_H_2_O concentration. CuSO_45_H_2_O was employed at five concentrations (100, 300, 500, 700, and 900 ppm Cu). The researchers reported that the control samples lacked significance in the 500–650 nm range, including the absence of CuNP production. According to reports, significant absorption peaks in the region above 700 nm for CuNPs were observed when the free-cell extract content in the resultant solution reached 700 and 900 ppm. The researchers detected a definite peak when about ten milliliters of extract free-cell was poured into 300 ppm of CuSO_45_H_2_O mixture, indicating that 10 mL of the upper phase was effective in reducing the copper ions in the combined mixture to generate copper NPs. Sadia et al. [113] found that the copper ion concentration affected the size of the nanoparticles, with the smallest typical size of NPs reported at lesser copper salt concentrations.

Shah et al. [114] revealed a spherical shape after adding 0.5 mM of copper sulfate salt. Akintelu et al. [111] determined that increasing the amount of copper salt caused bigger CuNPs.

Bhattacharya et al. [103] stated that at a lower concentration of 0.5 mM, there was a deficient development of Cu oxide nanoparticles. At a concentration of 1 mM, Cu oxide NPs with a size particle of 3.6 nm were created. However, at higher concentrations of Cu^2+^, the increase in the size of the particle was a result of the production of an advanced amount of atomic mass and their immediate development. The particle sizes for copper ions were 41.7 nm, 42.3 nm, and 43 nm, attained for concentrations of 1.5 mM, 2 mM, and 2.5 mM, individually. Hence, a concentration of 1 mM of CuSO_4_ was chosen.

Dlamini et al. [115] testified to the effects of concentration on the synthesized copper nanoparticles. The researchers found that the as-synthesized CuNPs were effective at a low concentration, with maximum flocculating activity attained at 0.2 mg/mL. The researchers also discovered that the concentration increases resulted in a decrease in activity. The authors noted that the excessive addition of flocculants destabilizes kaolin particle suspension, leading to the negatively charged repulsion of kaolin particles. This repulsion may be due to the excess flocculating agent inhibiting the site’s binding of the kaolin particles.

### 3.2. Effect of pH

Din and Rehan [116] stated that the length of NPs is affected by pH. In an acidic solution, smaller nanoparticles are formed, and as the pH of the mixture reaction increases, the NP size also increases. A significant growth in size was observed above pH 5. The authors further stated that copper nanoparticle oxides are produced at high pH due to the excess hydroxide. Nevertheless, several authors have documented the production of CuNPs at pH levels from 9 to 11. As the pH rises, the amount of hydroxide rises, resulting in the production of Cu OH^−1^. This describes how CuNPs are created in basic solutions [117].

Shende et al. [118] stated that in comparison to low pH values, higher pH values yield smaller nanoparticles. The discrepancy is caused by the rate at which Cu^2+^ ions are reduced by phytochemicals. CuNPs were not produced when a copper chloride (CuCl_2_) solution was added to *Dodonaea viscosa* extract. To fabricate CuNPs, the reaction mixture of the pH was adjusted to a basic standard. Din et al. [119] found that at low pH, larger-sized nanoparticles with rod or triangular shapes are produced, while at high pH, smaller-sized nanoparticles with spheres are produced. Similarly, Rajesh et al. [120] stated that when the pH rises from 6 to 10, the particle size is reduced by 18 to 9 nm. Nevertheless, as the pH surpasses 11, the particle diameter rises.

The size and texture of several formed NPs were impacted by the fluctuations in pH. Most of the time, the optimal pH for the NPs produced from a bioflocculant is 7–9 [112].

Sadia et al. [113] reported the mean NP size, noted at a low pH of 3.0 for the synthesized CuNPs. The increase in the pH was caused by the increase in the mean length of the NPs.

Hassabo et al. [121] found that altering the pH of the medium from 6 to 8 or increasing the temperature reaction caused the clustering of copper nanoparticles. Large particles of 74.5–108.1 nm were found.

### 3.3. Effect of Temperature

In general, heat is a crucial component in the fabrication of NPs of various sizes as well as shapes [122]. The optimum temperature recommended to produce nanoparticles using bioflocculants is 25–100 °C [123]. High temperatures also affect the morphological features of NPs. The time rate of incubation of the reaction medium has a large impact on the form, size, and stability of green-produced nanoparticles. In a study conducted by Mdlovu et al. [124], they examined the synthesis of copper nanoparticles at a constant temperature of 100 °C under continual stirring for 7–8 h. They concluded that at the ideal temperature, the process of structure formation occurred more rapidly, and the reduction of Cu^2+^ ions tended to occur within fully developed particles. Sadia et al. [113] detailed the influence of varying temperatures on the creation of NPs, as temperature increases result in an increased size of the nanoparticles.

Hong et al. [125] examined the impact of reaction heat on the formation of CuNPs. In the study, different temperatures such as 65, 70, 75, 80, 85, and 90 °C were investigated. They discovered that at a temperature of 65 °C, the reaction was not fully formed. Increasing the heat to 75 °C caused the formation of copper nanonuclei, but some mild aggregation was observed. Tiny and well-distributed particles were found at 80 °C. However, when the temperature surpassed 80 °C, a smaller copper nanoparticle size was observed with some aggregation. The findings align with previous studies, suggesting that at extreme temperatures, ethylene glycol acts as a covering agent, and the ethylene glycol OH base reacts with Cu to produce a protective coating, limiting the growth and aggregation of CuNPs [126]. Nevertheless, the increased temperature caused the stabilizers in the reaction system to lead to the agglomeration of CuNPs, which did not cover the nanoparticle’s surface [125].

### 3.4. Effect of Reaction Time on Copper Nanoparticle Production

Bukhari et al. [89] revealed that the time reaction is critical for the formation and stabilization of NPs. Researchers reported no color change in the synthesis of CuNPs after 60 min at an absorption of 550 nm. After 96 h, the CuSO_4_ solution was reported to be completely reduced to CuNPs by *Shewanella oneidensis* [127]. Ghorbani et al. [128] testified the formation of CuNPs using *Salmonella typhimurium* after 20 min.

Akintelu et al. [111] revealed that the quality, properties of morphology, and Cu nanoparticle yield are significantly affected by the length of incubation and time reaction. Reports showed that the features of the synthesized nanoparticles are also affected by dissimilarities in the incubation period and storage conjunction conditions [129]. The researchers stated that prolonging incubation periods causes aggregation and potential reduction in nanoparticle formation. Benassai et al. [130] stated that some nanoparticles occur at high reaction times. Pham et al. [129] conveyed the production of CuNPs via UV analysis to begin at 2 h and complete at 4 h. An additional increase in the reaction time to 3 h resulted in a rise in the strength of peak absorption, signifying the stability of the produced CuNPs.

## 4. The Characterization of Nanoparticles

Nanoparticles possess distinct physical, chemical, and biological characteristics that set them apart from bigger particles composed of the same material [131]. Scientists can analyze these properties through characterization, which allows them to manipulate and comprehend these unique traits [132]. This is particularly vital for creating novel materials and technologies. Additionally, characterizing nanoparticles is crucial for quality control purposes, as it helps ensure that the nanoparticles produced are consistent and safe for use [133]. Comprehending the toxicity and environmental impact of nanoparticles through characterization is also crucial. In addition, by analyzing nanoparticle properties, researchers can develop new applications and design nanoparticles with specific characteristics that are ideal for targeted uses, such as drug delivery and energy storage [133]. Some of the characterization techniques used in nanoparticles include scanning electron microscopy (SEM), Fourier infrared spectroscopy (FT-IR), transmission electron microscopy (TEM), X-ray diffraction (XRD), SEM-EDX, and the UV–Vis spectrum are discussed in detail below.

### 4.1. X-ray Diffraction (XRD) Analysis

X-ray diffraction (XRD) analysis is an effective tool for characterizing nanoparticles, including grain size, nature phase, and structural nature [76]. One of the key parameters calculated using XRD is the crystalline structure, which is assessed by utilizing the Debye–Scherrer equation. This equation utilizes the broadening of the maximum strong peak in an XRD measurement to calculate the crystalline structure [134]. By comparing the position and intensity of the peaks to reference patterns, the constituents of the particles can be determined. However, XRD is inappropriate for studying amorphous substances, as the peak produced by XRD for granules below three nanometers seems too big [135].

Dlamini et al. [45] reported that the XRD patterns of Cu nanoparticles, which were produced utilizing a bioflocculant, were compared to those of a standard copper sample (JCPDS 04-0836). The properties of peak diffraction for Cu were monitored at approximately 33° and 47° at a 2θ angle, corresponding to the (111) and (220) planes of the face-centered cubic (fcc) structure. The absence of peak impurity in the materials indicated that pure CuNPs were produced using the bioflocculant and copper salt. The moderate temperature during synthesis sharpened the diffraction peaks, indicating the growth and improved quality of the CuNPs. The XRD study depicted that the nature of the CuNP was crystalline and the intense Bragg reflections suggested that the bioflocculant used in the synthesis contributed to the strong X-ray scattering centers in the crystalline phase. Thus, the XRD outcomes displayed that the bioorganic phase of the NPs crystallized on the surface of the CuNPs. The widening of peaks in the XRD pattern was linked to the size particle effects, indicating lower-size particles. The larger peaks may represent the influence of laboratory instances on the nucleation and growth of crystal nuclei. The particle size was calculated using the Debye–Scherrer equation, revealing a crystalline range in the sizes of 6–10 nm, demonstrating an increase in surface area to volume ratio of the NPs.

According to Hong et al. [125], the properties of the CuNPs produced using ascorbic acid were investigated. The results portrayed the presence of three distinct peaks at 43.3, 50.4, and 74.08, which closely matched the Committee Joint on Diffraction Powder Standards of CJDPS XRD with accession number 00-004-0836, affirming the crystal structure of copper. To assess the solution of CuNP stability, the samples were kept, and a subsequent analysis revealed that the values of the three characteristic peaks remained unchanged. This observation confirmed that the samples could be stored for a minimum of 3 months while maintaining their integrity.

John et al. [79] outlined the XRD of the homogenized copper oxide NPs from *Brevundimonas* ef1. The authors observed the patterns’ diffraction at 2Ɵ of 32.42°, 35.47°, 38.59°, 48.66°, 53.54°, 58.69°, 61.70°, 66.01°, 68.18°, and 75.19° values. These values occurred to the corresponding (110), (002), (111), (112), (020), (202), (113), (310), (220), and (004) planes. The X-ray absorption examination demonstrated that the produced CuO NPs possessed a well-crystalline structure, specifically a structure monoclinic of CuO. This identification was supported and verified following the Committee Joint on Diffraction Power Standards (CJDPS) with the corresponding accession number of 89-5895.

Another study carried out by Duong et al. [136] utilized the XRD method to analyze the crystalline phase and properties of Cu/Cu_2_O. The XRD data indicate the existence of diffraction peaks at 2θ = 36.3, 42.2, 61.1, 73.2, and 77.1°, which match with the (110), (111), (200), (220), (311), and (222) lattice planes in the CuO structure, identified as CJDPS with the accession number of 05-0667. In addition, the author also reveals the presence of Cu at 2θ = 36.2, 42.6, 50.7, and 74.4°, with the corresponding lattice planes (111), (111), (200), and (220) of the face-centered cubic structure of copper zerovalent identified as CJDPS with accession number of 04-0836 The crystal average size of CuO and Cu was determined for XRD peaks at 36.3 and 42.6° using the Scherrer equation. Consequently, the crystal average sizes of CuO and Cu are approximately 40.5 and 28.3 nm, respectively.

### 4.2. Transmission Electron Microscopy (TEM) Analysis

One of the most often utilized practices for the classification of nanoparticles is TEM examination. TEM offers high-resolution images of the NPs, allowing for the evaluation of their size, design, and morphology. Additionally, by performing designated area electron diffraction (DAED) or high-resolution transmission electron microscopy (HRTEM), TEM may be used to investigate the structural crystallinity of NPs. The interaction of a thin material with a homogenous electron beam with an energy range of 60 to 150 kilo electron volts (KeV) is used in TEM tomography [137]. As the beam electron interacts with the material, a fraction of the electrons pass through and are transmitted, while the remaining ones undergo elastic or inelastic scattering [138]. By analyzing the data acquired from the transmitted electrons, the final image is generated [139].

Bhattacharya et al. [103] observed rodlike structures with clustered Cu oxide NPs, which appear to be incorporated in the matrix agal with the least agglomeration.

John et al. [79] studied the TEM micrograph of CuNPs using *Marinomonas* ef1. The authors revealed that the particles of the synthesized CuNPs are mono-dispersed, with spherical shapes and diameters that range from 10 nm to 70 nm. Tsilo et al. [48] detected a spherical-agglomerated CuNPs. The size particles of the formed CuNPs were estimated to be 20 nm.

Viet et al. [140] employed the TEM technique to see the sizes and shapes of copper nanoparticles. The researchers observed that the produced copper NPs exhibited a spherical form, and their sizes were consistently distributed within a range of 20 to 50 nm. Homogeneity in both size and shape was found to be associated with the specific copper phase utilized during synthesis.

### 4.3. Scanning Electron Microscope (SEM) Analysis

SEM is a widely used tool for finding detailed pictures of surfaces with high resolution. It is also applicable to studying materials at the nanoscale. By utilizing SEM in transmission mode, it becomes possible to analyze advanced nanoparticles, obtain comprehensive information about their properties, and examine groups of nanoparticles together [141]. When merged with energy-dispersive X-ray analysis (EDX), SEM proves to be a powerful method for evaluating the morphology and chemical composition of nanoparticles synthesized through green methods [142]. This combination allows for the generation of superior-quality images and enables a rapid assessment of the dimension and arrangement of the nanoparticles.

The investigation shown by Dlamini et al. [109] focused on the synthesis of CuNPs using a microbial bioflocculant obtained from *Alcaligenes faecalis* HCB2. The resulting CuNPs were observed to possess an amorphous shape and were found to be agglomerated. The particle typical size of these nanoparticles was measured to be 100 nm.

Bezza et al. [143] published SEM images of the created CuNPs employing *Bacillus cereus* SPL-4. Copper nanoparticles showed that the materials were uniform and well-dispersed. SEM-EDX analysis showed that synthesized the copper nanoparticles using bacteria were composed of pure copper and oxygen, signifying copper oxidation. The copper and oxygen weight composition were 88.50 and 11.50 (wt%), respectively, representing a stoichiometric ratio of Cu to O of 1.97:1, which is near 2:1, indicating that the compound produced is copper oxide (Cu_2_O) NPs. The appearance of other elements in the EDX spectra might be attributable to medium components or other additional molecules produced by the microbes. This result is congruent with that reported by Al-Qasmi [144] in the synthesis of CuNPs using chia seeds, which revealed the presence of copper and oxygen with 79.9 and 20.1 (wt%).

Sagadevan and Koteeswari [145] analyzed the morphology of copper nanoparticles using JEOL JSM-67001. The nanoparticles were established to be globular and had a size diameter of approximately 50 nm. The CuNPs’ alignment elements were found by employing the energy-dispersive X-ray (EDX) technique, and the corresponding spectrum. The existence of copper NPs was verified by the detection of strong peaks in the spectrum of EDX.

### 4.4. Fourier-Transform Infrared Spectroscopy (FT-IR) Analysis

FT-IR is a method used to analyze the functional groups and chemical bonds of the nanoparticles and their capping agents. It can also confirm the creation and stability of the NPs [120]. In FT-IR, infrared rays passed through the sample, with some being absorbed and the remainder passing through. The resulting spectra show the absorption and transmission characteristics of the sample material.

Guo [146] discusses the practice of FT-IR varieties analysis to investigate the components of MBFR10543 responsible for the elimination of Cu (II) from a liquefied medium. The Cu (II)- free FT-IR band showed numerous prominent and strong maxima at certain wave numbers, including the presence of carboxyl, hydroxyl, acetyl, and amide groups, with all of these performing a key role in Cu (II) elimination. The considerable alterations in the spectrum length and intensity of these distinctive maxima following Cu (II) resorption in the spectra of Cu (II)-loaded MBFR 10543 show that functional structures were primarily engaged in Cu (II) absorption into MBFR 10543. The Fourier-transform infrared study also revealed chemical occurrence, including hydroxyl, carboxyl, and amide functional groups that occur throughout the process of flocculant.

The Cu (ll)-free FT-IR spectra displayed numerous unique and strong peaks at certain wave numbers, indicating the occurrence of hydrogen, carboxyl, acetyl, and amine residues that were crucial in Cu (II) elimination. The considerable alterations in waveform length and the intensity of such particular bands following Cu (II) sorption within the spectrum of Cu (II)-loaded MBFR10543 imply that functional chains were primarily engaged in Cu (II) absorption onto MBFR10543. The chemical interactions with the carboxyl group, hydroxyl, as well as amine functional chains, were also seen throughout the process of flocculation, according to the FT-IR study.

Pavithran et al. [58] revealed that the FT-IR spectrum of biosynthesized copper NPs exhibited an extensive peak at 3294.38 cm^−1^ denoting the existence of O-H groups on the surface of the biosynthesized CuNPs. They also observed the peak at 2929 cm^−1^ that attributed to the symmetric C-H stretching vibration of the extract, while the peak at 163 cm^−1^ represented the non-reacted ketone group, suggesting the existence of flavanones adsorbed on the surface of biosynthesized CuNPs. The peaks at 1542 cm^−1^ and 1454 cm^−1^ showed the C-C stretch in aromatic rings.

In Ghosh et al. s’ [147] study, the infrared (IR) spectrum analysis of copper nanoparticles revealed specific groups at 1631 cm^−1^, 2225 cm^−1^, and 3305 cm^−1^. These bands were affiliated with multiple functional moieties that are found in NPs. Specifically, the group at 1631 cm^−1^ indicated the occurrence of a carbonyl group, while the band at 2225 cm^−1^ indicated C-O widening in alcohols. Additionally, the band at 3305 cm^−1^ was attributed to N-H primary aromatic amines and O-H groups in alcohols.

The FT-IR spectrum analysis of the CuNPs further indicated that the NPs were encompassed by various organic molecules, including terpenoids, alcohols, ketones, aldehydes, and carboxylic acids. These findings indicate the presence of a diverse range of organic compounds attached to the surface of the copper nanoparticles.

Noman et al. [148] reported that FT-IR of the produced copper NPs showed simultaneous peak absorption at different wavelengths, including an unstable band at 112.83 cm^−1^, and a complex protecting form. The band at 3396.44 cm^−1^ was a result of the hydroxyl (O-H) group of alcohol, while the bands at 2959.06 cm^−1^ were caused by C-H stretching. The absorption peaks at 1659.18 and 1451.44 cm^−1^ in the spectrum were attributed to the bending vibrations of the O-H group. Furthermore, the absorption peak observed at 618.53 cm^−1^ indicated the formation of Cu-O nanoparticles

According to Bhattacharya et al. [103], the group at 3406 cm^−1^ in the IR spectra was generated by the OH stretching of moisture. This is explained by the NPs’ larger surface-to-volume ratio of the nanoparticles and ability to absorb moisture. Table 2 below summarizes the FT-IR spectra of the synthesized CuNPs. The bands observed at 2857, 2927, and 3562 cm^−1^ were triggered by C-H and O-H stretching, individually [149]. The extension at 1087 and 1116 cm^−1^ matched the C-O curve of polymers present in algal biomass, such as flavones, terpenoids, and polysaccharides [150]. The stretching at 1634 cm^−1^ is linked to the C=O and N-H curves [150]. The appearance of C-O in the material may be caused by the widening at 882, 937, and 985 cm^−1^ [150]. The bands observed at 425, 486, 521, 602, 736, and 787 cm^−1^ matched the C-O curve [150]. The strong peak at 602 cm^−1^ was typical, indicating the production of the Cu-O bond in Cu oxide nanoparticles and the (1-01) direction [151]. The curve at 521 cm^−1^ was caused by the elongation of the CuO bond across the (101) path, as authenticated by the XRD line at 202. The curve established the generation of Cu oxide nanoparticles [151]. Thus, the occurrence of the various functional groups is significant for nanoparticle stabilization. The electrons’ doublet found in the homogenized CuNPs emphasizes the usefulness of CuNPs for the stabilization of electrostatic and was therefore employed as a protective agent.

### 4.5. UV–Vis Spectroscopy Analysis

UV–Vis spectroscopy is a popular approach for analyzing copper nanoparticles. The analysis involves measuring the absorption of light in the UV–Vis region of the electromagnetic spectrum [152]. Metallic nanoparticles can be formed from metallic salts, and their specific peaks at different absorptions can be observed by employing UV–visible spectrophotometry. Copper nanoparticles, for example, can be characterized by a visible color shift based on the surface plasmon resonance (SPR) concept. This is caused by the coupled resonance of the open electrons band in the metal oxides, which is energized by the incident UV light and induces surface plasmon absorption [152]. The changing color shift is caused by nanoparticles’ localized surface localized plasmon resonance (SLPR), which occurs in the observable portion of the electromagnetic spectrum [153]. The general peaks for synthesized copper nanoparticles in UV–visible spectroscopy are shown by strong distinctive peak absorption near 567 nm, which is detected for CuNPs in nano copper-loaded connected chitosan with fibers cellulose due to their surface plasmon resonance impact [129]. Other studies have also reported the presence of a peak around this wavelength for copper nanoparticles synthesized using different methods [69]. However, it is important to note that the peak may fluctuate liable on the synthesis method and mass of the NPs.

Tiwari and co-workers demonstrated the formation of CuNPs synthesized by the bacterium *Bacillus cereus*, which displayed surface plasmon resonance (SPR) maxima at wavelengths ranging from 570 to 620 nm and 350 to 370 nm. The particle size distribution varied from 11 to 33 nm, with a polydispersity index of 0.433. The CuNPs’ zeta potential was measured to be 9.6 mV [154].

Guru Bharathi et al. [155] found that CuNPs produced utilizing *Xenorhabdus* sp. reaction mixtures displayed a color shift from bluish to deep green. The solution’s UV–Vis absorption spectra portrayed a prominent peak absorption in the 335 nm band, indicating the formation of CuNPs. The average particle size of the synthesized CuNPs was discovered to vary between 5 and 20 nm, with clustered shapes.

Dlamini et al. [109] investigated the spectra of both bioflocculant and bioflocculant-synthesized CuNPs using UV–Vis spectroscopy. The experiment was carried out with a resolution of 1 nm in the distance range of 300–700 nm. The peak intensities for both the bioflocculant and the produced CuNPs were seen at about 280 nm in the plasmon resonance spectra, indicating the formation of CuNPs. The shift in the precise location of the SPR zone is affected by particle parameters such as shape, size, and protecting agents.

## 5. Comparison between the Antibacterial Activity of Copper and Silver Nanoparticles

Previous studies have primarily focused on utilizing bioflocculants to produce diverse types of metal NPs, for instance, silver and copper NPs, to combat microbial infections. These NPs have exhibited antimicrobial characteristics against both Gram-positive bacteria, like *Staphylococcus aureus*, and Gram-negative bacteria, such as *Escherichia coli* [156]. In addition to their efficacy against the aforesaid bacteria, these metals also affect the growth of *Bacillus subtilis*. Metallic nanoparticles have wide-ranging applications in various fields, including drug delivery, food, the purification of water, cosmetics, etc. [157]. Copper and silver NPs are frequently selected among the metal nanoparticles, since they are affordable and reliable in terms of physical and chemical properties, as well as possessing compatibility with polymers [158].

Research has compared the microbicidal effects of silver and copper NPs on various strains of microorganisms, precisely *E*. *coli*. *B*. *subtilis*, and *S*. *aureus*. The antibacterial capacity was measured, as well as the minimal growth inhibitory dosages (MICs) and lowest bactericidal dosages (MBCs). Among all the *S*. *aureus* strains tested, no significant differences in susceptibility to silver and copper nanoparticles were detected. Nevertheless, when contrasting *E*. *coli* with *S*. *aureus*, it was found that the alteration in reactivity was dramatically smaller for Ag nanoparticles than for Cu nanoparticles [56].

Nowak et al. [159] documented comparable results regarding the antibacterial properties of Ag and CuNPs against *E*. *coli* and *B*. *subtilis*. Their study revealed that Cu materials exhibited predominant antibacterial effects compared to Ag alloys. This observation aligns with other studies that indicated that CuNPs may have a stronger antimicrobial effect due to their ability to generate reactive oxygen species (ROS), which can damage bacterial cell membranes and disrupt intracellular processes more effectively than AgNPs [160,161]. Additionally, the size and morphology of CuNPs play a significant role; smaller nanoparticles possess a higher surface area-to-volume ratio, enhancing their reactivity and ability to penetrate bacterial cells more efficiently than larger AgNPs [162]. This synergistic effect is further supported by findings that combinations of Ag and Cu nanoparticles can exhibit enhanced antibacterial properties, suggesting complementary mechanisms of action between the two metals [163].

## 6. Antimicrobial Activity of Cu Nanoparticles

In the past few decades, the utilization of metal and metal oxide NPs in the treatment of bacterial and viral diseases has garnered major interest. Nanoparticles-based antibiotics and other drugs have gained remarkable attention because of their minimal toxicity, friendliness to the environment, and prospective effectiveness in treating diseases. Numerous studies have demonstrated the remarkable antimicrobial capabilities of Cu and Cu oxide NPs toward a variety of pathogenic microorganisms [164]. It has been observed that a high level of Cu nanoparticles is hazardous to a variety of bacterial diseases that affect both humans and plants [165]. Cu nanoparticles possess unique characteristics such as being tiny in size, with a large surface space, biological compatibility, and chemical responses, which contribute to their efficient eradication of bacterial cells.

The antimicrobial activity of biofabricated CuNPs against both Gram-positive and Gram-negative strains of pathogenic bacteria has been demonstrated [166]. Several studies have reported that CuNPs are promising and have high antimicrobial activities. These non-materials are either antibacterial agents for Gram-positive bacteria, including *Staphylococcus aureus* [32,33,167,168,169,170,171], *Bacillus subtilis* [32,168,172,173], and *Bacillus cereus* [170], or/and Gram-negative bacteria, including *Escherichia coli* [168,169,171,172,173,174,175], *Salmonella typhi* [64], *Klebsiella pneumonia*, *Enterobacter*, and *Micrococcus* [64]. Copper nanoparticle activity revealed positive marks with a defined inhibitory zone in comparison to conventional antibiotics such as Chloramphenicol [168], Streptomycin [64], Ampicillin [173], Cefepime hydrochloride monohydrate L-arginine [32], and antifungal drugs, including Fluconazole [32,168]. Therefore, the excellent antimicrobial activity produced by copper nanoparticles emphasizes its potential for usage in a diversity of practices, such as dentistry, wound repair, the nutrition industry, and wastewater treatment.

## 7. Application of Copper Nanoparticles

CuNPs possess a broad range of scientific applications and demonstrate notable efficacy against various harmful microorganisms. CuNPs induce the fabrication of oxygen-reactive species within bacterial cells, causing them to burst. Additionally, CuNPs have displayed promising capabilities as anticancer agents and antifungal agents. The antimicrobial properties of CuNPs have resulted in their utilization in food preservation and agricultural practices, protecting against detrimental fungi and bacteria [176]. In the realm of agriculture, Cu-based nano fertilizers and nano pesticides foster crop growth and nutrient levels. Moreover, Cu-based phytoremediation is important in wastewater purification and the elimination of thrash contaminants in the environment. Furthermore, owing to its excellent electrical conductivity, copper is employed as a superconductor and makes significant contributions to the field of modern electronics [177]. Some of the CuNP applications are detailed below.

### 7.1. Application of CuNPs for Wastewater Treatment

Cu nanoparticles have been widely studied for utilization in wastewater remediation [178]. Copper and its compounds are effective at eliminating a broad range of bacteria, including *Vibrio cholerae*, *Shigella* sp., *E*. *coli*, *Salmonella*, fungi, and viruses [179]. In hospitals, copper surfaces are utilized to prevent the growth of bacteria. By including fibrous materials that function as long-lasting reservoirs of Cu ions, the antibacterial and catalytic properties of CuNPs can be enhanced. CuNPs have also been proven to be useful in the application of cellulosic substances by researchers. The antimicrobial and catalytic properties of CuNPs can be improved by integrating fibrous materials that act as long-lasting reservoirs of copper ions. Researchers have also demonstrated the application of copper nanoparticles to cellulosic materials. Additionally, CuNP membranes can be utilized as antibacterial purifiers for drinking water [180]. Some more applications of copper nanoparticles are discussed below.

#### 7.1.1. The Removal of Pollutants by CuNPs Synthesized Using Microbial Bioflocculant and the Greener Method

Pollutants in wastewater must be removed to preserve human health and the surroundings and enable water reuse. Wastewater can contain harmful pollutants that can endanger human health if not properly treated. The phosphorus and nitrogen derived from human excretions, food, and certain soaps and detergents can contribute to nutrient pollution in local water bodies and harm aquatic ecosystems. Recent developments in the treatment of wastewater technologies have led to a high rate of removal of a variety of harmful contaminants, enabling water recycling across numerous industries [181].

As stated by Dlamini et al. [115], copper NPs can be utilized as a substitute for chemical flocculants because they are environmentally safe and readily demolished. The study found that copper nanoparticles were able to eliminate COD by 93% and BOD by 96% from coal mine effluent, which is higher than the removal effectiveness of polyamine flocculants, whereby the efficiency removal for COD and BOD were 89% and 73%, individually. Copper nanoparticles were also tested in the removal of phosphate and sulfate in comparison with polyamine flocculants. The authors found that copper nanoparticles showed a high removal efficiency of 85% for phosphate and 76% for sulfate, while polyamine revealed a removal efficiency of 76% for phosphate and 63% for sulfate. This suggests that copper nanoparticles have potential as an effective substitute for chemical flocculants in the treatment of wastewater applications. Table 3 shows the removal effectiveness of copper nanoparticles in mining water.

Bhagat et al. [88] utilized CuNPs to remove contaminants from effluent samples isolated from treatment plants’ water. The biosynthesized CuNPs are effective in removing pollutants such as phosphorus and sulfur, which results in eutrophication that affects aquatic life. Additionally, CuNPs have been used to eliminate contaminants from household wastewater industry and coal mines.

A study by Tsilo [182] detailed a significant investigation focused on the synthesis of copper nanoparticles using a bioflocculant derived from Kombucha tea SCOBY yeast. The synthesized CuNPs demonstrated remarkable removal efficiencies for various pollutants in wastewater. Specifically, they achieved a removal efficiency of 93% for chemical oxygen demand (COD) and 96% for biochemical oxygen demand (BOD) when applied to different water samples, including coal mine wastewater and domestic wastewater. These results indicate that CuNPs can effectively reduce organic matter in contaminated water, which is crucial for improving water quality and supporting aquatic life. In addition, the researcher revealed that these CuNPs were capable of removing nutrients such as phosphorus (P) and sulfur (S), which are often responsible for eutrophication in water bodies. The removal efficiencies for phosphorus and sulfur were found to be 52% and 83%, respectively.

In a recent study by Batool et al. [183], the bacterial synthesis of copper nanoparticles using a *Bacillus flexus* strain isolated from textile wastewater was reported. The researchers reported significant reductions in various pollutants, including chemical oxygen demand (COD), sulfates, and phosphates in textile wastewater. Specifically, the reduction percentages were approximately 39.659% for COD, 43.157% for sulfates, and 49.493% for phosphates.

Another study focused on the synthesis of copper oxide nanoparticles using *Hibiscus sabdariffa* leaf extract. The synthesized nanoparticles demonstrated significant effectiveness in reducing biochemical oxygen demand (BOD) and chemical oxygen demand (COD) by 56%. Additionally, the CuO NPs were found to effectively remove chromium (26%), copper (78.8%), and chloride (78.2%) from wastewater samples [184].

The research conducted by Nzilu et al. [185] utilized *Parthenium hysterophorus* aqueous extract to synthesize copper oxide nanoparticles, which were subsequently characterized and tested for their ability to degrade rifampicin in wastewater. The study revealed that these nanoparticles achieved an impressive degradation efficiency of 98.43% under optimal conditions, specifically at a pH of 2, a temperature of 65 °C, and a dosage of 50 mg.

The study conducted by Patel and Bhatt [186] assessed the effects of copper nanoparticles produced using *S. polyrhiza* on the removal of pollutants in wastewater, specifically focusing on chemical oxygen demand (COD) and biochemical oxygen demand (BOD). The researchers reported impressive removal efficiencies, with COD reduction measured at 55,263.3 ± 3298.5 mg/m^3^min and BOD reduction at 30,560.3 ± 1987.5 mg/m^3^min.

#### 7.1.2. Heavy Metal Reduction from Water

The elimination of heavy metals from wastewater is crucial for several reasons. For example, heavy metals such as lead, mercury, cadmium, and arsenic are highly toxic and can have severe environmental implications. They can contaminate water bodies, soil, and vegetation, leading to the disruption of ecosystems and the loss of biodiversity. Heavy metals pose significant risks to human health. If consumed through contaminated water, they can accumulate in the body over time and generate a variety of health concerns, such as organ damage, neurological diseases, developmental abnormalities in children, and even cancer [187]. There are regulatory standards and guidelines in place to restrict the dosage of heavy metals in wastewater, and compliance with these regulations is crucial to prevent the discharge of harmful pollutants into the environment. Removing heavy metals from wastewater is essential for water safety and ensuring that it meets the required standards for drinking, cooking, and other domestic uses [187]. Employing microbial bioflocculant- and bioflocculant-passivated nanoparticles for the elimination of toxic metals from effluent can help protect the surroundings, safeguard the health of humans, and maintain the integrity of water resources.

According to Gupta et al. [188], copper (11) oxide NPs can be employed to eliminate chromium from tannery sewer water. The researcher discovered that CuNPs with a medium size of 8 nm become more effective in eliminating Cr (IV). These nanoparticles have a crystal form and a mono structure. The researchers improved the relevant factors such as pH, dosage, contact duration, temperature, and starting Cr (IV) ion concentration using a batch adsorption approach. The outcomes demonstrated that Cu oxide nanoparticles were an efficient non-adsorbent for eliminating Cr (IV) ions from effluent. This reaction was optimized using a pH of 3, a Cr (IV) dosage of 20 mg/L, and an adsorbent dosage of 1.6 g/L. Other researchers have demonstrated that Cu oxide NPs may be utilized to eradicate contaminated metals from wastewater like Ni, Pb, Cd, and Cr (IV) [189].

As reported by Verma et al. [190], Cu oxide NPs are effective for removing Pb (11) and work as an excellent adsorbent. The absorption of lead onto CuO nanoparticles is an endothermic process and occurs spontaneously, which indicates a promising prospect of Cu oxide NPs for the elimination of lead from effluent.

In another study, Darwesh et al. [151] investigated the use of biosynthesized copper oxide nanoparticles immobilized in alginate beads to treat raw textile wastewater. They reported a significant reduction in microbial load, with a decrease of 99.995%, and heavy metal removal efficiencies of 93% for lead (Pb), 55% for chromium (Cr), and 30% for nickel (Ni). Additionally, dye removal efficiency reached approximately 90%, indicating the potential of CuO NPs in effectively treating textile effluents.

The study by Mahmoud et al. [191] focused on the green synthesis of environmentally friendly copper oxide nanoparticles using extracts from mint leaves and orange peels as reducing agents. The researchers investigated the removal efficiencies of Pb(II), Ni(II), and Cd(II) from contaminated water using CuO nanoparticles. The maximum uptake capacities (qm) for CuO NPs were found to be 88.80 mg g^−1^ for Pb(II), 54.90 mg g^−1^ for Ni(II), and 15.60 mg g^−1^ for Cd(II), with optimal conditions observed at a sorbent dose of 0.33 g L^−1^ and a pH of 6.

The aqueous extract of *Portulaca oleracea* was utilized to bio-fabricate copper oxide nanoparticles, which were subsequently tested for their ability to remove heavy metals from tanning wastewater [189]. The researchers reported significant removal efficiencies for various heavy metals under optimal treatment conditions. Specifically, the synthesized CuO NPs achieved removal rates of 73.2% for cobalt (Co), 80.8% for lead (Pb), 72.4% for nickel (Ni), 64.4% for cadmium (Cd), and an impressive 91.4% for chromium (Cr(VI)).

#### 7.1.3. Removal of Dyes

The elimination of colorants from wastewater holds significance in addressing these effects and guaranteeing the secure and enduring administration of water resources [192]. When dyes enter wastewater, they can cause several environmental effects and poor water quality [193]. They can persist in water bodies, alter light penetration levels, affect aquatic flora and fauna, and reduce overall water quality [194]. Industries release organic dyes into water streams, which is a significant source of environmental pollution. These dyes do not only pose chemical and aesthetic impacts but can also impede biological processes by disrupting light. Wastewater typically contains concentrations of dyes ranging from 10 to 200 mg/L [195].

Dlamini et al. [115] performed decolorization experiments using bioflocculant-synthesized copper nanoparticles to remove staining dyes for a solution. The process consisted of adding 1 mL of biosynthesized CuNPs to a 50 mL dye solution (4 g/L), stirring the solution for 1 min, and allowing it to rest for 10 min at ambient temperature. Safranin, methylene blue, carbol fuchsine, and malachite green were the dyes tested. After a minute of stirring and 10 min of settling, the supernatant was examined by employing the spectrophotometric UV–Vis. At the optimum spectrum of each dye, the reactivity of each sample was studied. The researchers reported that the produced Cu nanoparticles exhibited high removal efficiency for staining dyes, with removal efficiencies of 92%, 94%, 97%, and 85% for safranin, carbol fuchsine, malachite green, and methylene blue, respectively. These experiments demonstrate the prospects of bioflocculant-homogenized copper NPs in the elimination of dyes and suggest their high potential for industrial applications employing ascorbic acid as a reducing and protecting agent.

Fathima et al. [178] reported synthesized copper nanoparticles, utilizing L-ascorbic acid to treat textile wastewater. They found that biosynthesized copper nanoparticles demonstrated significant effectiveness in eliminating various dyes. The synthesized CuNPs were able to remove 91.53% of methylene blue, 84.89% of Congo red, and 73.89% of methyl red, respectively.

In a work conducted by Raina et al. [196], copper nanoparticles were detected to demonstrate significant effectiveness as catalysts in breaking down different types of organic dyes. The researchers observed that under ideal conditions, Copper NPs displayed remarkable catalytic activity in degrading methyl red (98.49%), methyl orange (98.84%), and phenyl red (99.62%).

In another study by Soomro and Nafady [197], CuNPs demonstrated excellent catalytic movement in the eradication of methylene blue (91.53%), methyl red (73.89%), and phenol red (not specified).

Zhang et al. [198] noted the completely degraded colors, namely methyl orange, phenol red, rhodamine b, and methylene blue, using the synthesized copper nanoparticles within 3 min.

Copper nanoparticles were produced utilizing a natural microbial strain of *Escherichia* sp. SINT7, according to Noman et al. [87]. The nanoparticles were constant and their sizes ranged from 22.39 to 39 nm. They found that the synthesized CuNPs were highly effective in removing azo dyes from textile effluent. The effectiveness for Congo red, malachite green, direct blue, and reactive black-5 at a dye dosage of 25 mg/L after 5 h of exposure were 97%, 90.55%, 88.42%, and 83.61%, respectively. The degradation percentages for the same dyes at a 100 mg/mL concentration were 83.90%, 31.08%, 62.3%, and 76.8%, respectively. The study also found that the treatment of textile effluents such as pH, electrical conductivity, turbidity, total dispersed solids, total liquefied solids, hardness, chlorides, and sulfates, in contrast to non-treated samples. This suggests that the use of biological synthesis of copper nanoparticles synthesized by *Escherichia* sp. SINT7 could result in the production of an environmentally approachable and affordable operation for large-scale wastewater purification.

CuNPs synthesized utilizing *Centaurea cyanus* plant extract demonstrated a remarkable ability to adsorb cationic dyes, achieving removal efficiencies of 95% for methylene blue and 90% for crystal violet under optimal conditions [199]. Therefore, these findings highlight the potential of CuNPs synthesized using biological procedures as effective agents in the treatment of industrial wastewater, particularly in mitigating the environmental impact of pollutants, heavy metals, and dye contamination from various dye solutions.

## 8. Mechanisms for Wastewater Purification from Heavy Metals and Dyes Using Copper Nanoparticles

Copper nanoparticles (CuNPs) are increasingly recognized for their effectiveness in removing heavy metals and dyes from wastewater through various mechanisms, primarily including adsorption, catalysis, and reduction. The high surface area-to-volume ratio of CuNPs enhances their interaction with pollutants, allowing them to effectively adsorb heavy metal ions and dye molecules onto their surfaces through electrostatic interactions and chemical bonding [200]. Additionally, CuNPs can act as catalysts in redox reactions, facilitating the conversion of harmful compounds into less toxic forms; for instance, they can reduce nitro compounds to amines or degrade organic dyes through oxidative reactions [201]. Furthermore, CuNPs can participate in reduction reactions where they donate electrons to transform toxic metal ions or degrade dye molecules into less harmful substances. To increase extraction efficiency, a preliminary functionalization of the nanoparticle surface is often performed, modifying CuNPs with various agents such as polymers or biomolecules to enhance their stability and interaction with target pollutants [202]. This functionalization introduces additional functional groups that improve the affinity of CuNPs for specific contaminants, thereby increasing their adsorption capacity and selectivity.

## 9. Toxicity of Copper Nanoparticles

Copper NPs have found diverse applications in lubricants, polymers, coatings, and metal inks. In lubricants, CuNPs enhance performance by acting as anti-wear additives, which can slightly decrease the friction coefficient while significantly reducing wear rates on metal surfaces [203]. Studies have shown that the optimal concentration of CuNPs in lubricating oils can achieve the best anti-wear performance, with reductions in friction coefficients observed under boundary lubrication conditions due to their unique rolling action at contact interfaces [204]. Moreover, CuNPs contribute to improved anti-corrosion properties by forming protective films on metal surfaces, mitigating oxidative wear and prolonging the lifespan of engine components. This film formation is attributed to the nanoparticles’ ability to create a barrier that prevents direct contact between metal surfaces, thereby reducing corrosion rates [203]. Additionally, the incorporation of CuNPs can enhance the thermal stability of lubricants, allowing them to perform effectively under the high-temperature conditions typically encountered in automotive applications [205].

While CuNPs offer significant benefits, there are rising concerns about their toxicity and ecological impact, necessitating further research on safe usage practices and methods to minimize adverse effects. Understanding the potential environmental and health concerns associated with the use of CuNPs in lubricants is crucial. Thus, it is essential to comprehend the detrimental consequences of CuNPs to evaluate the risks involved and ensure their safe utilization [56]. The following section provides a detailed account of some of these potential negative impacts.

### 9.1. Toxicity of CuNPs in Animals

Recent research has focused on examining how copper nanoparticles affect animals [206,207]. One certain study examined the influence of CuNPs preserved on titania on ovarian porcine cells [207]. The findings of this study indicated that copper nanoparticles had positive effects on ovarian granulosa cells, leading to enhanced cell proliferation, turnover, feasibility, and release of hormones. Consequently, this suggests that copper nanoparticles could serve as a safe alternative to toxic bio-stimulating agents in the reproductive processes of animals. However, another study explored the effect of CuNPs on the livers of rats and revealed that an excessive dosage of nano-copper can result in severe liver impairment, as evidenced by significant increases in levels of aspartate transaminase (AST) and alkaline phosphatase (ALP) [208]. It was shown that CuNPs can reach various biological areas, enter the bloodstream, accumulate in the liver, and induce oxidative stress, which is the primary cause of organ cytotoxicity associated with nano-copper. Hence, the effect of CuNPs on animals is determined by elements such as concentration, size, and other relevant considerations.

### 9.2. Toxicity of CuNPs in Humans

Exposure to nanoparticles by humans can occur through several main routes, namely inhalation, absorption through the skin, contact with the eyes, and ingestion [206,209]. When considering their size, nanoparticles have the potential to remain in lung tissue, causing increased oxidative stress and inflammation as a result of irritation. The researchers conducting the study discovered that copper nanoparticles measuring 23.5 nm in size exhibited greater toxicity than larger particles measuring 17 µm, primarily due to the nanoparticles’ enhanced ability to enter the body [210]. These individual copper nanoparticles can traverse between cells or penetrate cellular membranes, ultimately entering the bloodstream. Subsequently, the cardiovascular system plays a critical role in nanoparticle distribution throughout the body, starting from the initial point of exposure and leading to their accumulation in various organs.

Rodhe et al. [211] used the leukemic cell line HL60 to compare the toxicity levels of Copper NPs and soluble CuCl_2_. The findings show that Cu nanoparticles are more harmful than other chemicals. CuNPs produced greater amounts of ionic Cu than Cu oxide nanoparticles, implying that the produced hazardous was due to a composition of NPs and Cu^2+^. The study found oxidation of DNA, ROS intracellular, and impairment mitochondria within 2 h of exposure. The expiration of cells was eventually assessed to occur via necrosis.

Jiang et al. [212] found that Cu oxide NPs trigger autophagy in the cells of human cancerous breasts in a time-based and dose-related way, culminating in the creation of autophagolysosomes.

### 9.3. Toxicity Mechanisms

**Figure 6:**
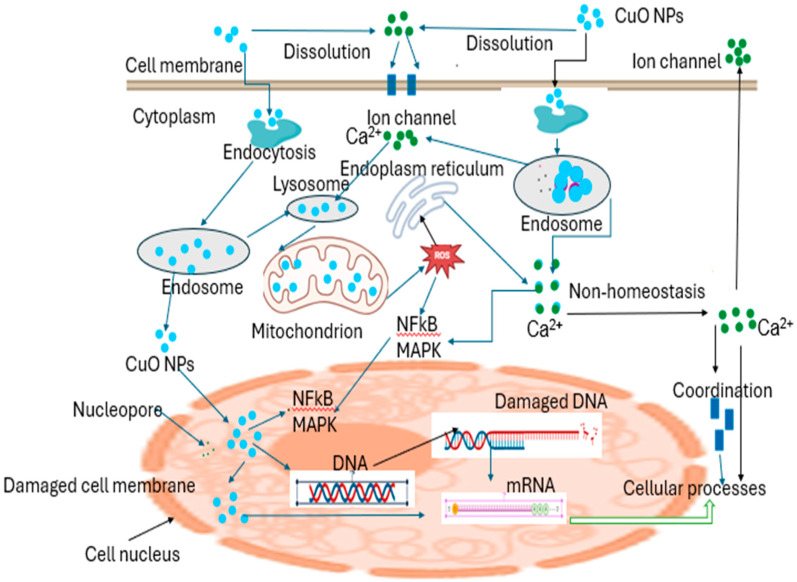
CuO nanoparticle mechanisms [56]. Created with BioRender.com. https://www.biorender.com (accessed on 27 April 2024).

According to Crisan et al. [56], the available literature on copper nanoparticles explores multiple ways in which they can be toxic. These mechanisms include free radical damage, DNA impairment, membrane disruption, mitochondrial impairment, the potential leakage of metal ions, and cessation. Figure 6 illustrates the mechanism of action for CuO nanoparticles. These mechanisms can result in various types of harm, such as increased lipid peroxidation, disrupted calcium and sulfhydryl homeostasis, and DNA damage. The reduction in glutathione and protein-bound sulfhydryl groups can lead to the formulation of reactive oxygen species like superoxide ions, hydrogen peroxide, and hydroxyl radicals. The oxidative stress caused by these processes can activate signaling networks associated with cellular dissipation and DNA impairment [56].

The production of reactive oxygen species (ROS) and the resulting oxidative stress are habitual mechanisms by which nanoparticles cause damage to cells. Cu oxide NPs can yield significant amounts of ROS, including O^2−^, OH, and H_2_O_2,_ even in small quantities [213]. When CuO nanoparticles enter the mitochondria, they disintegrate the membrane cell and promote the production of ROS. The dissolution of the particles is affected by numerous features including particle size, area surface, the composition of chemicals, pH, temperature, and organic matter [206]. If CuO nanoparticles penetrate nucleic acids, they can release Cu^2+^ ions, which contribute to oxidative damage and DNA damage. The oxidative stress resulting from these processes can cause DNA damage, lipid peroxidation, and the activation of signaling networks associated with cell death. The characteristics of CuO nanoparticles, such as their crystalline phase, adsorption ability, and solubility, also capacitate intracellular effects [206].

## 10. Methods for Reducing the Toxic Effect of Cu Nanoparticles on the Human Body

To mitigate the toxic effects of copper nanoparticles on the human body, various methods primarily centered around surface modification techniques can be employed. These strategies aim to enhance the biocompatibility of CuNPs while reducing their potential harmful effects. Some of the approaches are explained below.

### 10.1. Surface Modification Techniques

This method involves coating CuNPs with biocompatible polymers such as polyvinylpyrrolidone (PVP), polyethene glycol (PEG), or chitosan. This process involves synthesizing CuNPs and dispersing them in a solvent, followed by the addition of the chosen polymer to create a protective layer. This layer minimizes direct contact between the nanoparticles and biological tissues, thereby reducing toxicity.

### 10.2. Functionalization with Ligands or Biomolecules

Another approach is functionalizing CuNPs with specific ligands or biomolecules, such as amino acids or peptides. This enhances their biocompatibility and allows for the targeted delivery to specific cells. The process includes incubating the nanoparticles with selected ligands in a suitable buffer and purifying them to ensure that only those with bound ligands remain.

### 10.3. Controlled Release of Copper Ions

Controlling the release of copper ions is crucial for minimizing toxicity. This can be achieved through encapsulation techniques using biocompatible polymers or hydrogels, which stabilize the nanoparticles and regulate ion diffusion. Such methods help in preventing sudden spikes in copper ion concentration that could lead to toxic effects.

### 10.4. Targeted Delivery Systems

Employing targeted delivery systems by conjugating CuNPs with targeting ligands specific to cell receptors can significantly reduce systemic exposure and enhance therapeutic efficacy. This strategy not only minimizes potential toxicity but also improves the effectiveness of treatments involving CuNPs.

## 11. Conclusions and Future Perspectives

Nanotechnology has caught the attention of many scientists due to many benefits that include high surface area, several uses, stability under harsh environments, the simple and effective manipulation of constituents, improved interaction, and others. The synthesis of copper nanoparticles using microbial bioflocculants provides a naturally friendly, cost-efficient, and practical technique for wastewater treatment. Synthesized copper nanoparticles employing microbial bioflocculants have a high flocculation activity, a high removal efficiency for various pollutants, and antimicrobial properties that make them effective in killing bacteria and other microorganisms in wastewater. The use of microbial bioflocculants for synthesizing copper nanoparticles is a promising approach that can be easily quantified for industrial applications. This method can revolutionize the field of wastewater purification by offering a supportable and operative resolution for removing pollutants from effluent.

Unfortunately, there is limited information about the production of copper NPs utilizing bioflocculants from January 2015 to December 2023. There is even less on the synthesis using microbial bioflocculants. According to our knowledge, only three authors studied the fabrication of Cu nanoparticles utilizing microbial bioflocculants. With this review, we trust that further studies on the synthesis, characterization, and utilization of copper nanoparticles synthesized using microbial bioflocculants are needed to fill information gaps and also to encourage the implementation of biosynthesized Cu nanoparticles in the treatment of water. Beyond wastewater treatment, copper NPs partake in prospective uses in numerous areas, including anticancer as well as antimicrobial agents. The rising usage of copper NPs generates concern about their possible toxicity to the environment. Thus, further studies are needed to understand the possible environmental impacts of copper nanoparticles. In addition, encouraging wastewater purification facilities a circular economy, and energy sustainability is crucial for progressing forward with improved treatment technologies.

## Figures and Tables

**Table 1:** Various methods for the synthesis of copper nanoparticles (CuNPs).

Method Type	Examples	Key Features	Advantages	Disadvantages	Citation
Chemical Methods	Liquid-phase reduction, Hydrothermal, Electrochemical	Utilizes reducing agents like sodium borohydride, hydrazine	High yield, controllable particle size	Risk of oxidation requires careful handling	[110]
	Example: CuNPs synthesized using CuSO₄ and NaBH₄	Simple process, widely used	Simple equipment requirements	Potential toxicity of chemicals involved	[110]
	Example: CuNPs with PVP stabilization	Stable dispersions	Versatile and adaptable to various applications	Environmental concerns with some reagents	[56]
Physical Methods	Mechanical milling, Laser ablation, Physical vapor deposition	In the top-down approach, the bulk material is reduced to the nanoscale	Can produce uniform sizes	Often expensive and complex equipment	[30]
	Example: Laser ablation targeting bulk copper	High precision in size control	Minimal chemical use	Energy-intensive and may require vacuum conditions	[30]
Biological Methods	Green synthesis using plant extracts	Eco-friendly, utilizes natural reducing agents	Environmentally sustainable	Variability in yield and particle size	[111]
	Example: *Lantana camara* extract for CuNP synthesis	Biocompatible materials	Potential for novel properties	Slower synthesis rates compared to chemical methods	[30]

**Table 2:** Summary of some functional groups of the synthesized copper nanoparticles.

SI No	Frequency (cm^−1^)	Allocated Bond	Citation
1.	3406	-OH widening	[103]
2.	2857, 2927, and 3562	C-H and O-H stretch	[149]
3.	425, 486, 521, 602, 736, 787, 882, 937, 985, 1087, 1116, and 1634	Cu-O, C-O bond, C=O, and N-H bond	[150]
4	521 and 602	Cu-O bond along (101) direction	[151]

**Table 3:** Pollutant removal in wastewater by CuNPs [115].

Flocculant	Kind of Effluent	Kind of Contaminants in Water	Water Quality Before Treatment (mg/L)	Water Quality after Treatment (mg/L)	Removal Efficiency (%)
CuNPs	Coal mine water	Phosphate	2.00	0.3	85
		Sulfate	0.55	0.13	76
		Chemical oxygen demand (COD)	154	11.2	93
		Biological oxygen demand (BOD)	123.2	5.0	96
Polyamine flocculant		Phosphate	2.00	1.3	76
		Sulfate	0.55	0.32	63
		Chemical oxygen demand (COD)	154	32.4	89
		Biological oxygen demand (BOD)	123.2	23.6	73

## Data Availability

Not applicable.
